# Khat Chewing Habit among School Students of Jazan Region, Saudi Arabia

**DOI:** 10.1371/journal.pone.0065504

**Published:** 2013-06-11

**Authors:** Rashad Mohammed Alsanosy, Mohamed Salih Mahfouz, Abdelrahim Mutwakel Gaffar

**Affiliations:** 1 Family and Community Medicine Department, Faculty of Medicine, Jazan University, Jazan, Saudi Arabia; 2 Substance Abuse Research Center, Jazan University, Jazan, Saudi Arabia; Catholic University of Sacred Heart of Rome, Italy

## Abstract

**Background:**

The use of Khat leaves (Catha edulis) in Jazan, southwest of KSA, is prevalent among all segments of the population.

**Objective:**

This study was conducted to assess the prevalence and predictors of Khat chewing among intermediate and secondary school students of Jazan region.

**Methodology:**

A cross-sectional survey was conducted in late 2011 in Jazan region. A random sample of 3923 students was selected from 72 intermediate and upper secondary schools representing the different educational sectors of the region. A structured self-administered questionnaire was used for data collection. Descriptive statistics, a chi-squared test and logistic regression were performed to examine the prevalence, associations and predictors of Khat chewing.

**Result:**

The overall Khat chewing prevalence among students was 20.5% (95% C.I.: 19.27–21.79). The prevalence was significantly higher among males, at 33.1% (95% CI: 31.16–35.08), than among females 4.3% (95% C.I.: 3.39–5.31) (***P***<0.001). Univariate analysis revealed that gender, age, academic performance, friends’ smoking and Khat chewing, and students’ smoking status were associated with a significantly high risk of Khat chewing (***P***<0.001 for all). The multivariate logistic regression analysis suggested that the most important independent predictors of Khat chewing among the students in our sample were students’ smoking status (OR = 13.02, ***P***<0.001), friends’ use of Khat (OR = 5.65, ***P***<0.001), gender (OR = 4.62, ***P***<0.001), and friend’s use of tobacco (OR = 1.43, ***P***<0.001).

**Conclusion:**

A significant percentage of students chew Khat. The abuse of Khat is significantly associated with gender, peer influence, and cigarette smoking. Intervention programs are needed to create awareness among school students and to reduce the prevalence of the habit and its unfavorable consequences.

## Introduction

Khat is a well-known natural stimulant from the Catha edulis plant, a flowering evergreen tree or large shrub of the Celastracea family. The plant is known by different names in different countries: Qat in Yemen and Saudi Arabia, Khat in Ethiopia, Mirra in Kenya, and Qaad or Jaad in Somalia. However, in most of the literature, it is known as Khat [1], [2].

Various related factors are involved in the use of Khat. It is commonly used for social recreation and occasionally as a medicine. Khat is used by students for examinations and by drivers of motor vehicles, especially on long-distance journey. During the war, soldiers were given Khat to enhance their performance [3]–[5].

Khat chewing is a common habit among the population in the Jazan region. This may be due to its location; it is situated in the southern part of the Kingdom of Saudi Arabia, adjacent to Yemen [6]. The practice is deeply rooted in the socio-cultural traditions of all segments of the Jazan region, including school students. Although the government of the Kingdom of Saudi Arabia banned Khat chewing, it remains prevalent in the region. A recent comprehensive survey of Khat chewing was conducted in the region in 2006 and covered a representative sample of 10000 participants (school and college students). The study revealed that the overall prevalence of Khat chewing in the studied population was 21.4%, and 21.5% of school students were Khat chewers, compared with 15.2% of college students [6], [7].

Khat is related to many severe public health and social problems [8]–[15]. In addition to health problems, Khat use is associated with wasted time because a considerable amount of time is spent chewing Khat. Khat also affects the economies of families in Jazan because it is not cultivated locally and is imported from Yemen [6]. Another group of studies demonstrated a clear association between heavy Khat consumption and psychosis [16]–[22].

School-aged students are considered adolescents. Adolescence is a period of transition from childhood to adulthood during which individuals experience enormous physical, psychological, and sexual changes. The economic, social, cultural, and political environments in which people live can directly or indirectly influence these changes. During this critical stage of the life cycle, adolescents require information and services that are carefully tailored to meet their needs. The main objective of this paper is to investigate the prevalence of Khat chewing and related factors among intermediate and high school students of Jazan region. No previous study has investigated Khat chewing among school students at the intermediate level.

## Materials and Methods

### Study Design

An observational cross-sectional survey targeted students at both the intermediate educational level and high school students in Jazan region. The inclusion criteria for the study sample were full-time student status, enrollment in one of Jazan’s intermediate or higher secondary schools during the academic year 2011/2012, and age range of 13–21 years.

### Study Setting

Jazan (also called Gizan) region is one of the thirteen regions of the Kingdom of Saudi Arabia. It is located on the tropical Red Sea coast in southwestern Saudi Arabia. Jazan covers an area of 11,671 square kilometers, including some 5,000 villages and towns. One hundred islands are attached to it, including the largest island of Farasan. Jazan region runs along the Red Sea coast for almost 300 km. It is a highly populated state with a total population of 1.5 million [23].

### Sample Size and Design

The study proposed a representative sample of 4,100 students. This sample size was based on the previous large Khat survey conducted in 2005 (which found a 21.4% prevalence, 95% C.I., marginal error 2%, non-response rate of 10%, and design effect of 1.5). The sampling design adopted a three-stage cluster random sample depending on educational sector, schools, and classes. Two primary and upper secondary schools for both genders were randomly selected from the nine educational sectors of Jazan region. The sampling frames for the selection of the schools and the study participants were prepared in consultation with the Ministry of Education, regional education directorate. Systematic random sampling was used to select the target schools, and probability proportional to size sampling (PPS) was used to determine the number of students in the selected schools. Systematic random sampling was used to select target students from the classes within each school. The general education system in Saudi Arabia consists of three levels: primary for six years, intermediate for three years, and upper secondary for three years. There are two types of schools, Quranic (Islamic) and formal schools. In the general education system, all schools use the curriculum endorsed by the Ministry of Education, but some schools add Quranic recitation as a major subject. These schools are referred to as “Quranic.” We refer to the other schools as “formal” schools.

### Data Collection

A standardized questionnaire was used for data collection and was modified to suit the school student population. A pilot study was conducted with 160 students to fine tune the questions prior to the data collection. Following the pilot study, minor modifications were made to the original questionnaire.

The questionnaire involved demographic data on the students and family characteristics as well as students’ academic performance, Khat chewing patterns and frequency, age of initiation of Khat chewing, and other factors related to Khat chewing. The questionnaires were distributed and collected by health workers who were given training sessions on data collection prior to the beginning of the field work. The anonymity of the participants was emphasized, and confidentiality was strictly maintained on all collected questionnaires. Permission was obtained from the headmasters of the schools and the instructors of the selected classes to collect data during classes.

The following operational definitions were used: (a) non-Khat user: a person who had never used Khat in any form; (b) prevalence of ever chewing Khat: the proportion of the study population that had ever chewed Khat in their lifetime; (c) prevalence of current Khat chewing: the proportion of the study population that had chewed Khat within 30 days preceding the study.

### Statistical Analysis

To ensure data collection quality, a field work supervisor reviewed the submitted questionnaires daily and reviewed any errors or inconsistencies immediately. The data entry stage began immediately after the completion of the data collection. The data entry took place at Jazan Substance Abuse Research Centre (SARC) under the supervision of a data analysis specialist. The data were entered into Epi Info version 6 and transferred into SPSS version 17 (SPSS Inc, Chicago, IL, USA). Double data entry was conducted to ensure high-quality data. The data analysis involved descriptive statistics as well as inferential statistics. Simple frequency tabulation was utilized to provide a general overview of the data. The Khat chewing prevalence was presented with a 95% confidence level. Differences in proportions were compared for significance using the Z test, chi-squared test, or Fisher’s Exact test. Univariate analyses were conducted to identify factors associated with Khat use as a dependent variable and a set of explanatory variables. Variables that were significant (***P***<0.05) were included in the stepwise backward likelihood multivariate logistic regression. Adjusted odds ratios (ORs) and their 95% confidence intervals were reported. Hosmer-Lemeshow statistics were used to evaluate the goodness of the fit of the model. All statistical tests were two-sided, and a level of ***P*** *<*0.05 was used to indicate statistical significance.

### Ethical Issues

The study proposal and instrument were approved by Jazan University's review board (IRB), and voluntary informed oral consent was received from all students enrolled in the study. Prior to the beginning of the data collection, permission was obtained from the Directorate of Education in Jazan region. For students younger than fifteen years old, a formal letter and consent form were sent to the student’s father/caretaker before the data collection began. During the distribution of the questionnaires, students were told that the collected information would be kept anonymous, that participation was completely voluntary, and that they had the right to withdraw from the study at any time. Data were collected by health personnel (nurses) to ensure a high degree of confidence. Regarding the documentation of verbal consent, the top of the first page of the survey questionnaire included a box to be checked by students indicating their agreement to participate. The data were anonymized to protect participants, and no names were required. Students meeting the selection criteria from different classes were grouped into one class. In some schools and in the mosques, the students completed the questionnaire and submitted it to an unknown person (a health professional).

## Results

The response rate for the distributed questionnaires was 95.68% (3,923 from the target of 4,100 students). The remaining 4.32% were non-responders and participants with missing data. The mean age of the participants was 15 years (SD = 2.01). Students from formal education constituted 85% of the target students. Most of the sampled students (75.1%) were in the 15- to 19-year-old age group. The distribution of students according to class level (grades) showed that the third level accounted for 40.9%, followed by 32.3% and 26.8% for the second and first class levels, respectively. The distribution of students according to the mode of living indicated that 61.3% of the students were from urban areas, compared with 38.7% from rural areas. As planned during the sampling, approximately 57.3% of the students were from upper secondary schools, and 42.7% were from intermediate schools. The gender distribution showed that 56.3% of the students were males and 43.7% were females (Table 1).

**Table 1:** Background Characteristics of the Study Population.

Characteristics	Intermediate		Secondary		Total
Type of School	*Male*	*Female*	*Male*	*Female*	
** Quranic**	174(18.7)	224(29.9)	174(13.5)	17(1.8)	**589(15)**
** Formal**	756(81.3)	524(70.1)	1111(86.5)	952(98.2)	**3343(85)**
**Age Groups**					
** 10–15**	415(44.6)	450(56.1)	10(0.8)	27(2.8)	**872(22.2)**
** 15–19**	515(55.4)	328(43.9)	1199(93.3)	909(93.8)	**2951(75.1)**
** 20+**	–	–	76(5.9)	33(4.4)	**109(2.8)**
**Class Level (Grades)**					
** First**	209(22.5)	225(30.1)	355(27.6)	264(27.2)	**1053(26.8)**
** Second**	329(35.4)	221(29.5)	406(31.6)	314(32.4)	**1270(32.3)**
** Third**	392(42.2)	302(40.4)	524(40.8)	391(40.4)	**1609(40.9)**
**Mode of Living**					
** Rural**	298(32.0)	320(42.8)	543(42.3)	360(37.2)	**1521(38.7)**
** Urban**	632(68.0)	428(57.2)	742(57.7)	609(62.8)	**2411(61.3)**
**Total**	**930(100)**	**748(100)**	**1285(100)**	**969(100)**	**3923(1009**

As shown in Table 2, the overall Khat chewing prevalence among students was 20.5% (95% C.I.: 19.27–21.79). The prevalence was significantly higher among males, at 33.1% (95% C.I.: 31.16–35.08), than females, at 4.3% (95% C.I.: 3.39–5.31) (***P***<0.001). The prevalence of lifetime Khat chewing among students was 24.2% (95% C.I.: 22.9–25.57). The 38.8% prevalence for males (95% C.I.: 36.82–40.88) was also significantly higher than the 5.4% prevalence for females (95% C.I.: 4.39–6.53) (***P***<0.001). The current Khat chewing prevalence among intermediate school students was 16.2%, which was significantly lower than the prevalence among upper secondary school students of 23.7% (***P***<0.001). The same indicators were found to be significantly lower for Quranic students, at 15.4% compared with 21.4% for formal education schools (***P***<0.001). The current Khat chewing rate for rural students was 22.6%, which was significantly higher than the rate for urban students of 19.2% (***P***<0.05). The prevalence of ever chewing Khat was 19.9% in intermediate schools, which was significantly lower (***P***<0.05) than in upper secondary schools (27.4%).

**Table 2:** Prevalence of Khat Chewing among Students.

Category	Current Khat Chewers		Ever Khat Chewers	
	No %	95% C.I.	No %	95% C.I.
**Gender**				
Male	733(33.1)*	31.16–35.08	860(38.8)*	36.82–40.88
Female	73(4.3)	3.39–5.31	92(5.4)	4.39–6.53
**School Level**				
Intermediate School	272(16.2)*	14.52–18.05	334(19.9)*	18.06–21.88
Secondary School	534(23.7)	21.98–25.49	618(27.4)	25.62–29.30
**School Type**				
Quranic Schools	91(15.4)*	12.76–18.59	122(20.7)*	17.63–24.17
Formal Schools	715(21.4)	20.03–22.81	830(24.8)	23.39–26.32
**Mode of Living**				
Urban	462(19.2)*	17.62–20.78	551(22.9)*	21.22–24.57
Rural	344(22.6)	20.59–24.79	401(26.4)	24.21–28.63
**Total**	806(20.5)	19.27–21.79	952(24.2)	22.9–25.57

* Significant Difference (***P*** Value <0.05),

Table 3 illustrates patterns of Khat chewing among both male and female students. It is clear from the table that the majority of students chew Khat in their homes, followed by friends’ homes, with a significant difference between male and female responses (***P***<0.001). Almost 44.9% of students buy Khat directly from sellers; 47% of boys buy Khat, whereas 31% of girls said that an elder person gave it to them (***P***<0.001). When students were asked how easy it was to obtain Khat, the majority reported that it was somewhat easy, with no significant difference between males and females (***P***>0.05). Students did not present different responses regarding age at initial Khat chewing, when they normally chewed Khat, or their frequency of Khat chewing (***P***>0.05). However, there were significant differences between male and female responses regarding time spent in Khat sessions, with whom the respondents chewed Khat, and motives for Khat chewing (***P***<0.001).

**Table 3:** Pattern of Khat Chewing Habits among Students.

Characteristics	Male	Female	Total	P Value
**Place of Khat Chewing (n = 839)**				***P***<0.000
In my house	219(28.6)	41(56.9)	260(31.0)	
In my friend’s house	211(27.5)	15(20.8)	226(26.9)	
In school	11(1.4)	1(1.4)	12(1.4)	
In public places	82(10.7)	1(1.4)	83(9.9)	
On occasions	105(13.7)	1(1.4)	106(12.6)	
Other place	139(18.1)	13(18.1)	152(18.1)	
**Do you think that it is easy to obtain Khat? (n = 834)**				***P*** *>0.050*
Yes	292(38.8)	27(33.3)	319(38.2)	
Yes to some extent	334(44.4)	41(50.6)	375(45.0)	
No	127(16.9)	13(16.0)	140(16.8)	
**Source of Khat (n = 777)**				***P***<0.000
From sellers	332(47.0)	17(23.9)	349(44.9)	
I give money to another person to buy it	45(6.4)	5(7.0)	50(6.4)	
I take Khat from another person	126(17.8)	13(18.3)	139(17.9)	
An elder person gives me Khat	84(11.9)	22(31.0)	106(13.6)	
Other	119(16.9)	14(19.7)	133(17.1)	
**Age at first time chewing Khat (n = 821)**				***P*** *>0.050*
Less than 10 years	57(7.5)	9(14.5)	66(8.0)	
10–14 years	380(50.1)	26(41.9)	406(49.5)	
Less than 19 years	322(42.4)	27(43.5)	349(42.5)	
**Time of Khat Chewing (n = 834**)				***P*** *>0.05*
Noon	93(12.2)	9(13.0)	102(12.2)	
Afternoon	97(12.7)	9(13.0)	106(12.7)	
Evening	66(8.6)	8(11.6)	74(8.9)	
Night	509(66.5)	43(62.3)	552(66.2)	
**Frequency of Khat Chewing (n = 780)**				***P*** *>0.050*
Daily	92(12.7)	10(18.2)	102(13.1)	
Most week days	92(12.7)	5(9.1)	97(12.4)	
Weekends only	288(39.7)	23(41.8)	311(39.9)	
On occasions	253(34.9)	17(30.9)	270(34.6)	
**Time spent in Khat sessions (751)**				***P***<0.000
One hour	90(12.9)	17(33.3)	107(14.2)	
2–4 hours	175(25.0)	21(41.2)	196(26.1)	
More than 5 hours	435(62.1)	13(25.5)	448(59.7)	
**With whom do you chew Khat (n = 841)**				***P***<0.000
Family	45(5.8)	23(32.9)	68(8.1)	
Relatives	152(19.7)	13(18.6)	165(19.6)	
Friends	464(60.2)	16(22.9)	480(57.1)	
Alone	110(14.3)	18(25.7)	128(15.2)	
**Reasons for Khat chewing (n = 380)**				***P***<0.000
Feel stressed	69(9.1)	15(20.5)	84(10.1)	
Anxiety	44(5.8)	1(1.4)	45(5.4)	
Need to change mood	356(47.0)	37(50.7)	393(47.3)	
Other reasons	288(38.0)	20(27.4)	308(37.1)	

Variables such the student’s age, academic achievement, pocket money, peers’ smoking, students’ smoking status and Khat chewing, probation during recent academic years, and depression were evaluated to determine the factor/s that may be associated with Khat chewing (***P***<0.001 for all) (Table 4).

**Table 4:** Comparison of Khat Chewers versus Non-Chewers with Regard to Some Variables.

Variables	Khat Chewing Status		P Value
	Khat Chewers	Non-Chewers	
**Age Groups**			***P*** *<0.001*
10–15	119(12.5)	753(25.3)	
15–19	784(82.4)	2167(72.7)	
20+	49(5.1)	60(2.0)	
**Academic Performance**			***P*** *<0.001*
Excellent	304(31.9)	1598(53.6)	
Very Good	371(39.0)	968(32.5)	
Good	250(26.3)	390(13.1)	
Pass	15(1.6)	19(.6)	
Fair	12(1.3)	5(0.2)	
**Pocket Money**			***P*** *<0.001*
Less Than 100 SR	181(25.8)	643(27.2)	
101–150 SR	104(14.8)	598(25.3)	
151–500 SR	264(37.7)	881(37.3)	
More Than 500SR	152(21.7)	243(10.3)	
**Student’s Smoking Status**			***P*** *<0.001*
Yes	489(54.3)	138(5.1)	
No	411(45.7)	2590(94.9)	
**Friends’ Smoking**			
Yes	592(66.0)	689(24.9)	***P*** *<0.001*
No	305(34.0)	2083(75.1)	
**Friends’ Use of Khat**			***P*** *<0.001*
Yes	746(81.2)	762(26.8)	
No	173(18.8)	2080(73.2)	
**Received probation during the past year?**			***P*** *<0.001*
Yes	293(34.6)	526(19.2)	
No	554(65.4)	2207(80.8)	
**Feel Depressed**			***P*** *<0.01*
Yes	404(46.2)	1397(52.0)	
No	470(53.8)	1289(48.0)	

The results of the univariate and multivariate logistic regression analyses for potential risk factors of Khat chewing are shown in Table 5. The univariate analysis revealed that gender, age, academic performance, friends’ smoking and Khat chewing, and students’ smoking status were associated with a significantly high risk of Khat chewing (***P***<0.001 for all). The multivariate logistic regression analysis suggested that the most important independent predictors of Khat chewing among the students in our sample were students’ smoking status (OR = 13.02, ***P***<0.001), friends’ use of Khat (OR = 5.65, ***P***<0.001), gender (OR = 4.63, ***P***<0.001), and friends’ use of tobacco or Khat (OR = 1.43, ***P***<0.001). School level, age, and depression were insignificant in the multivariate logistic regression.

**Table 5:** Univariate and Multivariate Logistic Regression Analyses for Khat Chewing-Related Factors among Study Participants.

Category	Univariate			Multivariate#		
	OR	95% C.I.	*P*. Value	OR	95% C.I.	*P*. Value
**Gender**						
Female (Ref.)	1			1		
Male	11.21	8.94–14.06	***P*** *<0.001*	4.63	3.15–6.79	***P*** *<0.001*
**School Level**						
Intermediate (Ref.)	1			1		
Secondary	1.52	1.31–1.77	***P*** *<0.001*	0.96	0.71–1.31	***P*** *>0.05*
**Age Group**						
10–14 (Ref.)	1			1		
15–19	5.17	3.38- 7.90	***P*** *<0.001*	1.82	0.79–4.21	***P*** *>0.05*
20+	2.26		***P*** *<0.001*	1.27	0.61–2.65	***P*** *>0.05*
**Academic Probation**						
No (Ref.)	1			1		
Yes	2.22	1.87–2.63	***P*** *<0.001*	1.43	1.08–1.91	***P*** *<0.05*
**Pocket Money**						
Less Than 100 SR(Ref.)	1			1		
100–150 SR	2.22	1.71–2.87	***P*** *<0.001*	1.35	0.91–2.00	***P*** *<0.001*
151–500 SR	3.60	2.69–4.81	***P*** *<0.001*	2.28	1.47–3.52	***P*** *<0.05*
More Than 500SR	2.09	1.63–2.67	***P*** *<0.001*	1.71	1.18–2.47	***P*** *>0.05*
**Student’s Smoking**						
No (Ref.)	1			1		
Yes	22.3	18.10–27.71		13.02	9.55–17.77	***P*** *<0.001*
**Friends’ Smoking**						
No(Ref.)	1			1		
Yes	5.87	4.97–6.91	***P*** *<0.001*	1.43	1.15–2.21	***P*** *<0.05*
**Friends’ use of Khat**						
No (Ref.)	1			1		
Yes	11.77	9.78–14.16	***P*** *<0.001*	5.65	3.923–8.15	***P*** *<0.001*
**Feel Depressed**						
No (Ref.)	1			1		
Yes	1.26	1.08–1.47	***P*** *<0.001*	1.13	0.87–1.47	***P*** *>0.05*

#Hosmer-Lemeshow goodness of fit test χ2 = 2.50 P = 0.976.

## Discussion

The present study aimed to update knowledge about the Khat chewing habit and its related factors among school students of Jazan region. The overall prevalence of Khat chewing among the students in our sample was estimated at 20.5% (33.1% for males and 3.4% for females). The results seem to agree with a recent large-scale study conducted in 2006 [6], [7], [24], [25], [26], which found an overall prevalence of Khat chewing of 21.1% (37.7% for males and 3.6% for females). These rates do not differ significantly from our findings. The same study indicated that the Khat chewing prevalence in rural and urban areas was 20.5% and 24.5%, respectively. Our analysis found that the estimates for rural and urban areas were 22.6% and 19.2%, respectively. Thus, the two estimates seem to be very close. Importantly, when considering the time dimension, it is clear that Khat chewing rates remained constant during this five-year period, suggesting that Khat awareness campaigns are not affecting Khat use in Jazan region.

The prevalence of Khat chewing was found to be 15.4% among Quranic (religious) schools, significantly lower than the 20.4% prevalence for formal education schools. This may be due to religious instruction in these schools because Khat is banned in Islam. There is a link between religion and adolescent behavior, such as smoking and risky behavior. Social scientific studies have shown that religion is often a positive influence in the lives of adolescents [27], [28].

Generally, the prevalence of Khat chewing in this study seems to be lower than studies conducted in other countries. The Khat chewing prevalence in Yemen, the country from which Khat is imported to Jazan region, is universal among all segments of the population. The WHO estimates that up to 90% of adult males and 50% or more of young women adopt the habit of chewing Khat. The same source estimated that 15–20% of children under the age of 12 are daily consumers of Khat [29]. A study from southwestern Ethiopia [30] found that the prevalence of Khat chewing among upper secondary school students was 64.9%. A survey conducted in a rural community in southern Ethiopia found a prevalence of 50% [31]. Another study conducted in Somalia reported that approximately 36.4% of the respondents had chewed Khat in the week preceding the interview [32].

One third of students reported that they chewed Khat in their homes, with a significant difference between males and females. This finding is similar to previous studies [7], [25], [26] and implies that any strategy for Khat prevention should start in the home. Although Khat is banned in Saudi Arabia, 45.0% of the study participants reported that it is somewhat easy to obtain Khat, and 45% bought Khat directly from sellers, indicating its availability in the region. Almost 50% of the students stated that they tried Khat for the first time between the ages of 10 and 15. This pattern is similar to previous studies conducted in Jazan and Ethiopia [7], [25], [26], [33]. Our study suggested that Khat is often chewed for a period of more than five hours (60%). This finding is comparable to other studies [34], [35] that found that Khat chewing sessions extend for more than five hours. Traditionally, Khat chewing used to be an afternoon activity (2–4 pm), but our study found that 66.2% of the participants chewed Khat at night. The consequence of this pattern of abuse is sleep disorders for most of the night that affect the next day's activities. A possible explanation of this new pattern is that students may avoid using Khat during the day.

The present study found that peer influence was an important factor in students’ practice of substance use. We found that students who reported that their friends used Khat and smoked cigarettes were more likely to be users of either one or both of the above substances than students whose friends were non-users. Previous studies have found that friends’ and parents’ use of substances is strongly associated with the use of substances among students, indicating the influence of peer pressure [36], [37], [38]. Our findings also indicate that students who reported cigarette smoking were more likely chew Khat than other students. Pocket money was also significantly associated with Khat use.

Perhaps the most important advantage of this study is that it included intermediate students for the first time. Similar to most behavioral studies, this study is not without limitations. First, the study used a descriptive single cross-sectional design that cannot establish trends or causality between substance use and potential risk factors. Second, the data were collected based on students’ self-reports and may be subject to recall bias and under-reporting of substance use due to social desirability bias. Finally, the report of lifetime Khat chewing in the current study is not the most sensitive indicator.

### Conclusions and Recommendations

This study found that a considerable magnitude of substance use among students. The findings of our study are similar to the results of a study conducted five years earlier, indicating that no change occurred during this period. This study found that substance use is significantly associated with gender, age, academic performance, and friends’ substance use. Cigarette smoking is also significantly associated with Khat use. Understanding the factors associated with Khat use is the first step in designing and implementing comprehensive anti-Khat use interventions that prevent multiple risk factors among intermediate and upper secondary school students. Traditional intervention programs are not sufficient; there is an urgent need for friendly intervention programs, such as peer education. Campaigns also must be implemented to create awareness among students and to reduce the prevalence of the habit and its unfavorable social and health consequences.
